# Pulmonary granulomatous inflammation after ceritinib treatment in advanced ALK-rearranged pulmonary adenocarcinoma

**DOI:** 10.1007/s10637-022-01270-2

**Published:** 2022-06-21

**Authors:** Juanyan Liao, Hui Guan, Min Yu, Ping Zhou, Yao Han, Xingchen Peng, Shuang Zhang

**Affiliations:** 1 Department of Biotherapy, Cancer Center, West China Hospital, Sichuan University, Sichuan Chengdu, China; 2 Department of Thoracic Oncology, West China Hospital, Sichuan University, Chengdu, Sichuan China; 3 Department of Pathology, West China Hospital, Sichuan University, Chengdu, Sichuan China; 4 Department of Respiratory, West China Hospital, Sichuan University, Chengdu, Sichuan China

**Keywords:** Non-small cell lung cancer, Anaplastic lymphoma kinase rearrangement, Ceritinib, Pulmonary granulomatous inflammation

## Abstract

Ceritinib is a new anaplastic lymphoma kinase (ALK) inhibitor that has shown greater potency in patients with advanced ALK-rearranged non-small cell lung cancer, including those who had disease progression in crizotinib treatment. Here we reported, after several months of ceritinib treatment, two patients with advanced ALK-rearranged pulmonary adenocarcinoma exhibited a spectrum of respiratory symptoms like cough and dyspnea, with significantly higher inflammatory indicators. Chest computed tomography (CT) showed multiple bilateral and peripheral lesions in lungs. The prior considerations taken into account were disease progression or infection. However, biopsies of the pulmonary nodules revealed features of granulomatous inflammation without definite cancer cells. We documented for the first time that ceritinib might be associated with pulmonary granulomatous inflammation, and clinicians should be alert to the possibility that the rare adverse event emerged during ceritinib treatment.

## Introduction

Lung cancer ranked first in cancer-related deaths and first in the new cases in males and third in females. In 2020 the number of new cases was 2,206,771 (11.4% of all cases), and the number of deaths was 1,796,144 (18% of the total) [1]. Anaplastic lymphoma kinase gene rearrangement occurs in 2–7% of non-small cell lung cancer (NSCLC) patients, especially in young, female, adenocarcinoma, non-smoking patients [2]. Ceritinib, a second-generation oral ALK inhibitor, was highly active in ALK-rearranged NSCLC patients including those who had resistance to crizotinib. Ceritinib could cause a series of adverse events, but pulmonary injuries are rare [3].

Herein, we reported that two patients with advanced ALK-rearranged pulmonary adenocarcinoma were treated with ceritinib for several months, and developed respiratory symptoms and pulmonary lesions. It was initially suspected of disease progression or infection, but turned out to be ceritinib associated pulmonary granulomatous inflammation, which was confirmed by pathological examination.

## Case report

**Fig.1:**
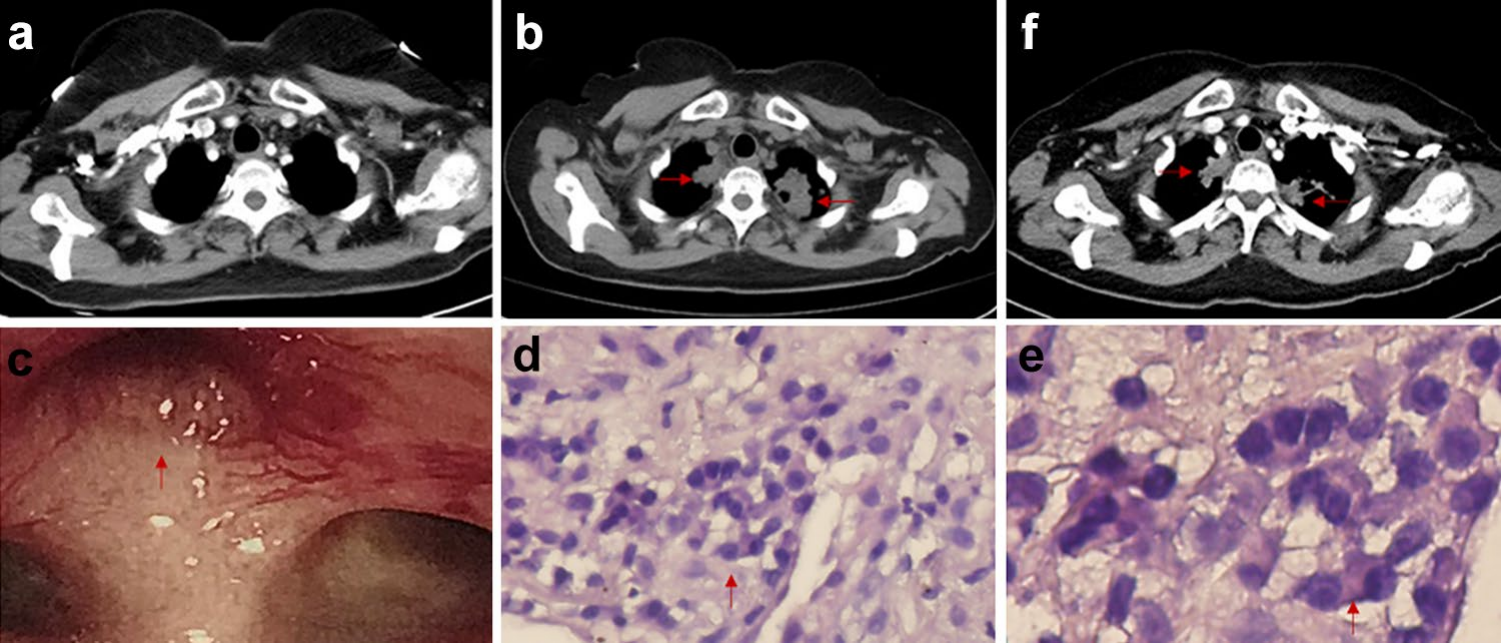
Computed tomography scan at baseline (**a**), bilateral pulmonary shadows after 6 months of ceritinib treatment (red arrows) (**b**), fiberoptic bronchoscopy showed narrowing of the right apical bronchus (red arrow) (**c**), the biopsy pathology demonstrated local granuloma formation (red arrows). Microscope magnification: 200 × for d, microscope magnification: 400 × for e (**d**-**e**), decreased bilateral lung shadows after 1 month of supportive treatment (red arrows) (**f**)

**Fig.2:**
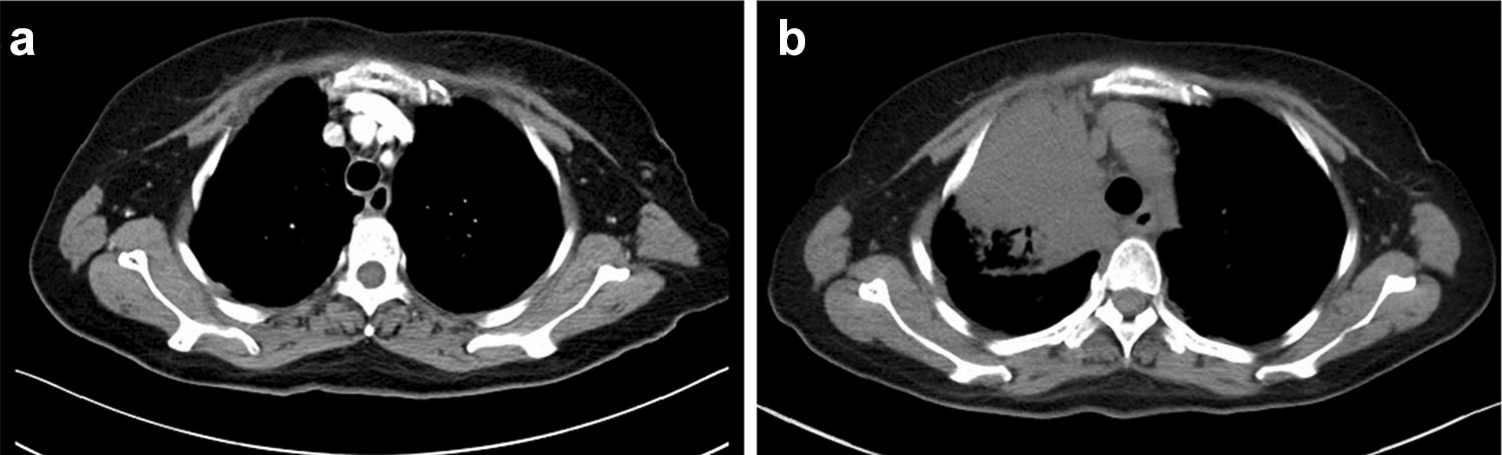
Computed tomography scan in ceritinib withdrawal (**a**), and disease progression in January 2020 in patient 1 (**b**)

In July 2015, a 60-year-old woman was diagnosed with stage IV lung adenocarcinoma with ALK gene rearrangement. The primary cancer was located in right upper lung lobe, and tumor metastatic site included bones and thoracic lymph nodes. Crizotinib at 250 mg twice daily was initially administrated for more than 3 years, and crizotinib was interrupted and discontinued due to multiple brain metastases detected by head MRI. The patient received subsequent treatment of ceritinib 450 mg daily. However, cough and dyspnea appeared without fever after 6 months of ceritinib therapy. Laboratory examinations were performed, and the results showed both C-reactive protein and erythrocyte sedimentation rate increased while sputum acid fast staining, sputum culture of mycobacterium tuberculosis, and interferon gamma release assays (T-SPOT assay) were all negative. The evidence of infection was not found. The chest CT scan showed new patchy shadows in bilateral lungs, which might be associated with tumor progression (Fig. 1a-b). Fiberoptic bronchoscopy showed swelling of the right upper bronchial mucosa, exposure of submucosal blood vessels, and narrowing of the apical bronchial lumen (Fig. 1c). The short period from ceritinib therapy to the symptomatic disease that was entirely unlike the reported mean disease control time, and relative literature indicated that pathological examination was proposed [4–6]. CT-guided lung biopsies were subsequently performed, and there were only chronic inflammatory cell infiltration and local granulomatous reaction without malignant cells in biopsy tissue (Fig. 1d-e). Diagnosis of pulmonary granulomatous inflammation was made according to the pathological results. After supplemental oxygen and antitussive and expectorant drugs, symptoms such as cough and dyspnea significantly improved soon and lumps in bilateral lungs were reduced or eliminated in October 2019 (Fig. 1f). Thereafter, ceritinib restarted from then and discontinued in January 2020 for cough and dyspnea, which were due to disease progression (Fig. 2a-b). She died of disease progression 4 years after initial treatment.

**Fig. 3:**
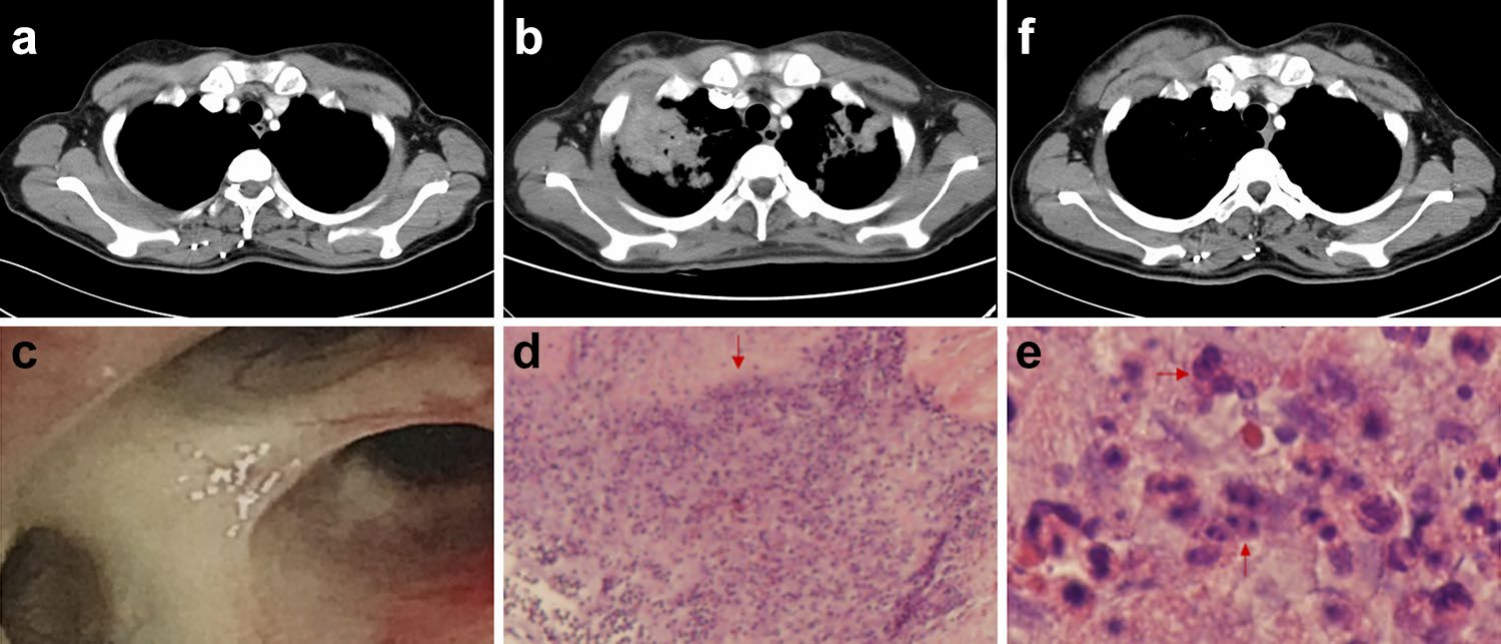
Computed tomography scan at baseline (**a**), bilateral pulmonary shadows after 6 months of ceritinib treatment (red arrows) (**b**), fiberoptic bronchoscopy showed no stenosis of the right bronchus (**c**), the biopsy pathology showed local granuloma formation and multinucleate giant cell infiltration (red arrows). Microscope magnification: 200 × for, microscope magnification: 400 × for e (**d**-**e**), bilateral lung shadows disappeared gradually after stop taking ceritinib (**f**)

**Fig.4:**
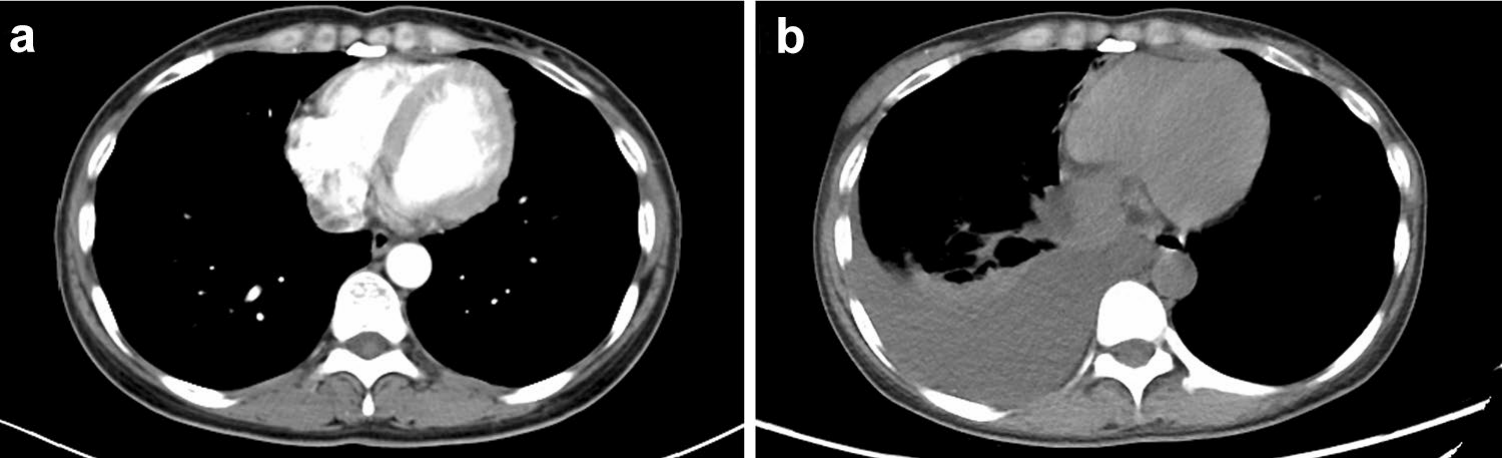
Computed tomography scan in ceritinib withdrawal (**a**), and disease progression in September 2019 in patient 2 (**b**)

In another 42-year-old woman, stage IV lung adenocarcinoma with ALK gene rearrangement in left lower lob was diagnosed in March 2017. Crizotinib was given as first-line treatment for six months until jaundice appeared. After liver function gradually improved and became normalized three weeks later, the treatment was switched to 450 mg ceritinib daily. Ceritinib was well tolerated for around 6 months, and interrupted by cough and fever in April 2018. We evaluated the patient with a clinical suspicion of infection or disease progression. The blood routine examination results showed an increase in white blood cells (10.19 × 10^9^/L) and neutrophils (7.98 × 10^9^/L), and pathogen detection including blood culture, sputum culture, fungi staining and culture, and acid-fast staining were all negative. Meanwhile, the chest CT result indicated multiple masses in bilateral lungs (Fig. 3a-b). The differential diagnosis included infection, disease progression, interstitial lung disease, or others. Bronchoscopy and biopsy were performed and no stenosis was found in the bilateral bronchus (Fig. 3c). Biopsy results demonstrated inflammatory exudation and local granuloma formation without evidence of malignancy (Fig. 3d-e). In pathological specimens, no positive bacilli were detected by acid-fast staining, no fungi were detected by silver hexamine and periodic acid Schiff reaction (PAS), and quantitative Polymerase Chain Reaction (qPCR) tests did not reveal the DNA fragment of tuberculosis. After excluding other possible causes of pulmonary lesions, clinical diagnosis of ceritinib-associated pulmonary granulomatous inflammation was made. After withdrawal of ceritinib, the patient was treated with oxygen inhalation, antibiotics and glucocorticoid. Result of laboratory tests showed elevated markers of inflammation were decreased. The clinical symptoms were gradually relieved and the chest CT result showed lung lesions became absorbed in bilateral lungs in September 2018 (Fig. 3f). The patient then received a sequence of bigatinib, systemic chemotherapy and bevacizumab. However, 16 months after withdrawal of ceritinib, the patient was hospitalized for disease progression and received best supportive care in September 2019 (Fig. 4a-b), and died several months later.

## Discussion

Granulomatous inflammation is a chronic proliferative inflammation characterized by granulomatous formation, which is involved in various systems such as the lung, skin, gastrointestinal tract, pelvis, muscles [7]. There are many causes of granulomatous inflammation, including infectious and non-infectious factors. Granulomatous drug eruptions are rare and include four major types: interstitial granulomatous drug reaction, drug-induced accelerated rheumatoid nodulosis, drug-induced granuloma annulare, and drug-induced sarcoidosis [8]. Most related drugs include antiretroviral therapy, tumor necrosis factor alpha antagonist, interferons, and immune checkpoint inhibitors. The drug-induced sarcoidosis reaction (DISR) often occurs in the respiratory system and skin [9–13]. In our two cases, the granulomatous reactions located in lungs. Before granulomatous reactions are diagnosed, other differential situations need to be ruled out, such as mycobacterial infections and tumors. Drug-induced granulomas generally resolved after interruption of the drug, which is the key difference from other causes.

Ceritinib is an oral, second-generation ALK inhibitor for the treatment of patients with ALK-positive, advanced NSCLC, especially those who are resistant to crizotinib. Ceritinib was 20 times more potent against ALK than crizotinib in a vitro enzymatic assay [14–17]. Experiments in mice showed that ceritinib is active against cancers derived from patients who have acquired resistance to crizotinib and is effective against ALK-positive cancers with resistance mutations of L1196M and G1269A [14]. Many clinical trials have demonstrated safety and efficacy of ceritinib in patients with ALK-positive NSCLC, and most toxicity were reduced or eliminated by dose adjustment [18, 19]. Hitherto, the common adverse events of ceritinib were slight or moderate gastrointestinal reaction including nausea and vomiting, diarrhea, elevated transaminase [2, 18, 20]. Lung toxicity is a rare but potential fatal side effect in NSCLC patients receiving ALK-inhibitors. A review concluded that lung toxicity including pneumonitis and interstitial lung disease occurred in 105 of 4943 patients (2.1%) [21] and other studies reported lung toxicity was 3.2% in patients treated with ALK-inhibitors [22]. Pulmonary adverse event during therapy with crizotinib, brigatinib, ceritinib, alectinib, and lorlatinib occurred in 1.8%, 7%, 1.1%, 2.6%, and 1.8% of the patients, respectively. 65% of cases are grade 3—4 adverse events, with a mortality rate of 9% [21]. In some clinical trials, total 1.0%-4.0% of patients appeared lung injuries treated with ceritinib [2, 20]. Therefore, we should take care when pulmonary symptoms appeared during the treatment of ceritinib. However, sometimes it was difficult to distinguish drug-related pulmonary adverse events from infection or disease progression. For example, one study reported a solid lung nodule like disease progression in a NSCLC patient treated with loratinib, while it was finally confirmed by biopsy as nodular granulomatous lymphadenitis [4]. In our cases, the nodule and shadows of lung in CT were ultimately proven to be grannlomatous inflammation caused by ceritinib, which was confirmed by pathology reports.

In our experience, the diagnosis of ceritinib-related pulmonary granulomatous inflammation was not specific and based on the exclusion of infections and tumor metastases in the lungs. In the two cases, both patients were hospitalized for respiratory symptoms with elevated infection indicators and lung shadows in chest CT. Prior diagnosis were infection or disease progression. However, combining medical history and other negative pathogenic examinations, drug-related reaction might be possible. Biopsy might be the proper method to distinguish benign and malignant lesions, and it indeed turned out to prove our suspicion. Our report suggested that physicians ought to be careful when patients appeared pulmonary symptoms with lung shadows mimicking progression during administration of ceritinib or other ALK-inhibitors, and drug-related inflammation should be under consideration. Once the diagnosis is established, corresponding measures should be taken as soon as possible. Mild-to-moderate respiratory symptoms could be relieved through dose adjustment, symptomatic treatment, such as supplemental oxygen, and antibiotics. Whereas for persistent or recurrent granulomatous inflammation, steroid hormones and immunosuppressants should be given and discontinued ceritinib if necessary [23].

## Conclusion

In conclusion, our report firstly displayed ceritinib-associated granulomatous pneumonia in ALK-NSCLC confirmed by pathology. Although ceritinib is effective and well tolerated, close monitoring of potential adverse effects especially lung injury was absolutely warranted. When NSCLC patients have respiratory symptoms such as cough and dyspnea while taking ceritinib, which could not be explained by lung infection or tumor progression, physicians should be alert to the possibility of ceritinib-related granulomas.

## Data Availability

The datasets generated during and/or analyzed during the current study are available from the corresponding author on reasonable request.

## Abbreviations

ALK: Anaplastic lymphoma kinase
NSCLC: Non-small cell lung cancer
CT: Computed tomography
DNA: Deoxyribonucleic acid
PAS: Periodic acid Schiff reaction
qPCR: Quantitative Polymerase Chain Reaction
DISR: Drug-induced sarcoidosis reaction
